# Time Outdoors at Specific Ages During Early Childhood and the Risk of Incident Myopia

**DOI:** 10.1167/iovs.16-20894

**Published:** 2017-02

**Authors:** Rupal L. Shah, Yu Huang, Jeremy A. Guggenheim, Cathy Williams

**Affiliations:** 1 School of Optometry and Vision Sciences, Cardiff University, Cardiff, United Kingdom; 2 School of Optometry, Hong Kong Polytechnic University, Kowloon, Hong Kong; 3 School of Social and Community Medicine, University of Bristol, Bristol, United Kingdom

**Keywords:** myopia, refractive error, time outdoors, ALSPAC, epidemiology

## Abstract

**Purpose:**

Time outdoors during childhood is negatively associated with incident myopia. Consequently, additional time outdoors has been suggested as a public health intervention to reduce the prevalence of myopia. We investigated whether there were specific ages during early childhood when the time outdoors versus incident myopia association was strongest.

**Methods:**

Children participating in the Avon Longitudinal Study of Parents and Children (ALSPAC) were studied from age 2 to 15 years. Parentally reported time outdoors and time spent reading were assessed longitudinally in early childhood (ages 2, 3, 4, 5, 7, and 9 years). Noncycloplegic autorefraction was carried out longitudinally in later childhood (ages 10, 11, 12, and 15 years). Information was available for 2833 participants. Cox proportional hazards regression was used to test for association between time outdoors and incident myopia.

**Results:**

From 3 years of age onward, greater time outdoors was associated with a reduced risk of incident myopia. The hazard ratio for myopia changed progressively from 0.90 (95% CI 0.83–0.98, *P* = 0.012) at age 3 years, to 0.86 (95% CI 0.78–0.93, *P* = 0.001) at age 9 years, for each additional SD of time spent outdoors per day. These associations were independent of two major risk factors for myopia: time reading and number of myopic parents.

**Conclusions:**

Additional time spent outdoors across the 3 to 9 years age range was associated with a reduced incidence of myopia between ages 10 and 15 years. There was a trend for the association to increase toward the older end of the 3 to 9 years range.

Myopia is a global problem with increasing prevalence rates reported across the world and reaching epidemic levels in urban areas of East Asia.^1–4^ Myopia poses a significant financial and social burden on individuals due to the on-going cost of optical correction, lowered self-esteem, and reduced participation in recreational activities.^5,6^ Furthermore, myopia is a leading cause of irreversible visual impairment and blindness; for example, in a recent study of Chinese adults aged 45 to 59, myopic macular degeneration was the leading cause of visual impairment.^7^

Refractive error is a complex, multifactorial disorder influenced by both genetic and environmental factors.^1,8,9^ Using an assumption-free method, genetic factors were found to explain approximately 30% of the variance in refractive error in a sample of 15-year-olds of European ancestry from the United Kingdom.^10^ Importantly, however, heritability estimates for refractive error vary widely,^11–13^ and genetic factors cannot explain the rapid increase in the prevalence of myopia over the past one to two generations.^1^ Environmental risk factors, such as the amount of time spent reading, are associated with the risk of incident myopia in some populations, although not all,^14–18^ and recent evidence has suggested a causal relationship between education and myopia,^9^ which builds on consistent epidemiologic evidence linking them.^1,19,20^ The time children spend outdoors is also associated with incident myopia.^21–23^ This relationship appears to be unrelated to near work tasks, because time outdoors remains strongly associated with incident myopia after controlling for time spent on nearwork activities, and because the time children spend in these activities tends not to be correlated.^21,22^ Furthermore, randomized controlled trials (RCTs) have demonstrated a reduced incidence of myopia in children allocated additional time outdoors during the school day, suggesting a causal relationship.^24,25^

With the evidence from RCTs that time outdoors has a protective role in childhood against incident myopia, two key questions now are: “Over what age range is time outdoors protective?” and “Are there specific ages at which time outdoors is most effective?” The only definitive way to address these questions would be to randomly assign groups of nonmyopic children to additional periods of time outdoors at one or more specific ages across early childhood, subsequently following their refractive development through to adulthood. However, recruiting and monitoring such a large sample of infants and children over 1 to 2 decades would pose serious logistic challenges. Here, as an alternative strategy, we used existing data from a large sample of children for whom time outdoors was ascertained prospectively from the age of 2 years, and whose refractive development was followed from age 7 years through to age 15 years, to investigate whether the natural variation in the time children spent outdoors at specific ages in early childhood was associated with a reduced risk of incident myopia in later childhood. This allowed us to test the hypothesis that there were critical ages at which spending time outdoors was most protective against incident myopia.

## Methods

The research adhered to the tenets of the Declaration of Helsinki. Participants were from the Avon Longitudinal Study of Parents and Children (ALSPAC), a population-based birth cohort study.^26^ Pregnant women with expected delivery dates between April 1, 1991 and December 31, 1992, residing in the former county of Avon, United Kingdom, were eligible for enrollment. This cohort consisted of 14,541 pregnancies, from which there were 13,988 children alive at 1 year of age. All participants provided informed consent. Ethical approval for the study was obtained from the ALSPAC Ethics and Law Committee and the Local Research Ethics Committees. The ALSPAC Web site contains details of all the data that are available through a fully searchable data dictionary (http://www.bris.ac.uk/alspac/researchers/data-access/data-dictionary/).

### Questionnaires

Questionnaires that included items relating to the amount of time their child spent outdoors during a typical day at that moment in time were distributed to mothers/primary carers at six time points when the participating children were aged approximately 2, 3, 4.5, 5.5, 6.5, and 8.5 years old (Supplementary Table S1). Weekdays and weekends were considered separately in these questions, with additional consideration of season and holidays added to the questionnaires at older ages. For example, at ages 6.5 and older, there were six questions following the format: “On a [school weekday/weekend day/school holiday day], how much time on average does s/he spend each day out of doors in [summer/winter]?” In most questionnaires, and for all those administered from when the child was aged 4.5 years, the response options for the amount of time spent outdoors per day were: “none at all,” “less than 1 hour,” “1 to 2 hours,” and “3 or more hours” (Supplementary Table S1). Respondents were asked to choose the duration category that matched the child's habits at that time.

To make the fullest possible use of the time outdoors data collected, the approximate number of hours per day each child spent outdoors was estimated at each of the six age points. This calculation required two simplifying approximations/assumptions to be made. First, categoric responses were converted into quantitative units (hours) as described in Supplementary Table S1. For example, the category “1 to 2 hours per day” was assigned a value of 1.5 hours per day. Note that this approach was inexact because the categories were not equally sized or spaced, and because the last category was truncated; for example, the category “3 or more hours per day” was assigned a value of 3 hours per day. Second, it was assumed that summer and winter each lasted for 13 weeks per year at each age point, with holidays accounting for 6 and 3 weeks of these summer and winter periods, respectively. The number of hours spent outdoors on week days and weekend days was converted to that for an average day using the formula:

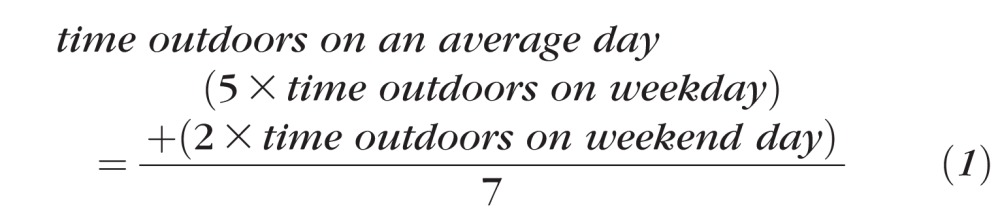



Converted exposures spanning summer, winter, and holiday periods at a particular age point were combined and means taken to give an estimate of average daily time outdoors across the year. For some analyses, time outdoors exposures were transformed to *z* scores (i.e., mean = zero, SD = 1) at each question age, to standardize questionnaire responses. A correlation matrix comparing outdoor exposures between each age point was generated using the “corrplot” package in R.

The myopia status (myopic versus not myopic) of the child's parents was estimated from the ALSPAC self-completion questionnaires sent during pregnancy. Both parents were asked the following question for each eye individually: “How would you rate your sight without glasses?” Response options were as follows: “always very good,” “I can't see clearly at a distance,” “I can't see clearly close up,” and “I can't see much at all.” Parents with both eyes categorized as “I can't see clearly at a distance” or “I can't see much at all” or a combination of these two responses were classed as being myopic in this study. Parents with both eyes categorized as “always very good” or “I can't see clearly close up” or a combination of these two responses were classed as being nonmyopic. Any other combination of responses resulted in the classification being set as “missing.” (Note that this classification scheme differed from that used in previous studies of refractive development in ALSPAC participants, as detailed in the Supplementary Information; the new classification scheme resulted in fewer parents' myopia status being classed as “missing.”)

From the age of 4.5 years, the questionnaires used to gauge time spent outdoors also included questions on the amount of time the child spent reading per day. The time spent reading questions followed the same format to questions on time outdoors (Supplementary Table S1). For example: “On a [school weekday/weekend day/school holiday day], how much time on average does s/he spend each day reading books for pleasure?” Response categories were the same as for time spent outdoors at the same age and were transformed to hours per day *z* scores using the same method.

### Refractive Error

An estimate of the child's mean spherical equivalent (MSE) refractive error was obtained using noncycloplegic autorefraction (Canon R50 instrument; Canon USA, Inc., Lake Success, NY, USA) at clinic visits scheduled at ages 7, 10, 11, 12, and 15 years. Outlier autorefraction values were removed as described previously.^27^ The estimated refractive error in the two eyes was averaged, and children with an average MSE ≤−1.00 diopter (D) were classified as “likely myopic,” whereas children with an average MSE >−1.00 D were categorized as “likely nonmyopic.” Cycloplegic autorefraction is considered the gold standard method for measuring the refractive error of children^28^; the implications of not using cycloplegia are considered in the Discussion section. At each clinic visit, the child's precise age was recorded.

### Cox Proportional Hazards Regression

Survival analysis using Cox proportional hazards regression models was undertaken using R and the “survival” package^29,30^ to test whether the amount of time spent outdoors during early childhood was associated with the relative risk (hazard ratio [HR]) of incident myopia between the ages of 10 and 15 years old. Survival analysis, unlike logistic regression analysis, has the advantage of being robust to the common occurrence of “right censoring” within the current dataset. This occurred when the likely myopic/nonmyopic status of a child was unknown due to nonattendance at one or more research clinics after being classified as likely nonmyopic at an earlier visit. Right censoring also occurred for children classified as likely nonmyopic at their final scheduled clinic visit at age 15 years, as their future myopia status was unknown but had the potential to change to likely myopic.

The child's age at their last clinic visit or when they were first declared as likely myopic, whichever occurred first, was used as their “survival time” for this analysis, with “survival” defined as remaining likely nonmyopic. Children were excluded from the survival analysis if their parentally completed questionnaire data were incomplete (i.e., if time outdoors or time reading information was not available for one or more of the age points) or if the child was already categorized as “likely myopic” at the age 7 refractive error assessment visit. A total of 2833 children were taken forward for this analysis. Univariate analysis was conducted using a model in which the only dependent (explanatory) variable was the amount of time spent outdoors at a particular age. To evaluate the extent of confounding by three key covariates in the above relationship between time outdoors and likely myopia, a multivariate analysis was performed as described for the univariate analysis but with the inclusion of either (1) number of myopic parents and sex, or (2) number of myopic parents, sex, and time spent reading.

This method of survival analysis requires the assumption of proportional hazards to be met. This assumption states that the hazard function of a covariate on event probability (for example, the risk of becoming likely myopic dependent on the amount of time spent outdoors at age 8.5 years) does not change over the observation period (for example, during the series of research clinic visits).^31,32^ In other words, the difference in the risk of becoming likely myopic at age 10 years between those with a 1 SD difference in time outdoors exposure would be similar to that at age 15 years. To verify that this assumption was met, Schoenfeld residuals were plotted against time for each covariate in addition to the overall model. This was performed for all three models at all questionnaire ages.

### Linear Mixed Models

Analyses also were carried out to examine how children's time outdoors behavior differed between those who later became myopic versus those who remained nonmyopic, using similar methods to those used previously by the Collaborative Longitudinal Evaluation of Ethnicity and Refractive Error (CLEERE) study group to address this question.^21^ Specifically, the amount of time spent outdoors at ages 2 to 8 years was modeled as the outcome variable for children who were versus were not classified as likely developing incident myopia in the future (between the ages of 10 and 15 years old). Linear mixed modeling was used for this analysis because it appropriately handles those with missing data regarding time spent outdoors at one or more ages, and takes into consideration the tendency for time spent outdoors between adjacent visits to be correlated. Children not already classified as “likely myopic” at the 7-year clinic visit and who had information available regarding their number of myopic parents and their time outdoors for at least one age point were included in this analysis, as long as they either (1) were classified as “likely myopic” at one or more refractive error assessment clinic visits between the ages of 10 and 15 years, or (2) attended the 15-year clinic visit and were classified as “likely nonmyopic” at that visit (and any previous visit). Age, myopia status, sex, number of myopic parents, and time spent reading were included as fixed effect explanatory variables in the models. In addition, the model fit was compared before and after the inclusion of one or more interaction terms for the above explanatory variables (e.g., age × myopia status; age × sex) to explore whether the “slope” of the time outdoors versus age relationship was different between boys and girls, or between children who did or did not go on to develop likely myopia during the follow-up period. Higher-order age terms (e.g., age^2^, age^3^) were also evaluated in the model, as fixed effects, to assess whether these relationships varied nonlinearly across childhood. The repeated measures of time outdoors, coded as *z* scores, were taken as the outcome variable for the models (fitted in the model as a random effect). The best-fitting model was determined by performing ANOVA analysis of competing models. The model with the lower log likelihood statistic was selected if the ANOVA comparison *P* value was below 0.05. Linear mixed modeling was undertaken using the R package “nlme.”^33^ Plots were generated using the R package “ggplot2.”^34^

Standard linear mixed models make the assumption that the outcome variable (here, time outdoors) has a normal distribution. This assumption was violated in our analyses, because time outdoors estimates were restricted to a finite number of values by the nature of the conversion from categoric response options (Supplementary Fig. S2A–F). This effect was most extreme for time outdoors ascertained at age 2 years, when rather than being spread across a wide distribution, most time outdoors estimates occurred at exactly 1 of 4 values (Supplementary Fig. S2A). Therefore, a sensitivity analysis was carried out using a mixed model in which time outdoors was fitted as a longitudinally sampled ordinal response (with four levels of response), using the R package “mixor.”^35^ Time spent outdoors at each age was converted from its pseudo continuous status to an ordinal variable with four levels, as shown in Supplementary Figure S2G–L. The model parameters obtained from the best-fitting linear mixed model were included in the mixor ordinal model to allow a direct comparison between the two classes of model.

## Results

**Figure 1:**
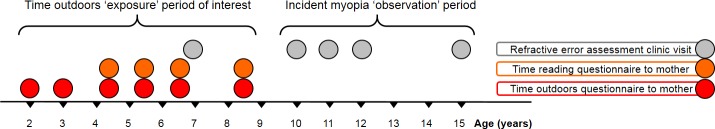
Time line showing age of child when questionnaires were administered and when refractive assessments were carried out.

Figure 1 summarizes the age points in early childhood at which information was collected about time spent outdoors and time spent reading, and the age points in later childhood at which refractive error was assessed. Table 1 summarizes the demographic characteristics of the 2833 children who had full information available for the “risk-factor” variables, sex, time spent outdoors at all six age points, time spent reading at all four age points, and the number of parents affected by myopia. Children who became likely myopic in the future typically spent less time outdoors at ages 3.0 to 8.5 years compared with those who remained likely nonmyopic (Table 1). The opposite trend was observed for time spent reading, with children who were to become likely myopic in the future typically spending more time reading than those who remained likely nonmyopic (Table 1).

**Table 1:**
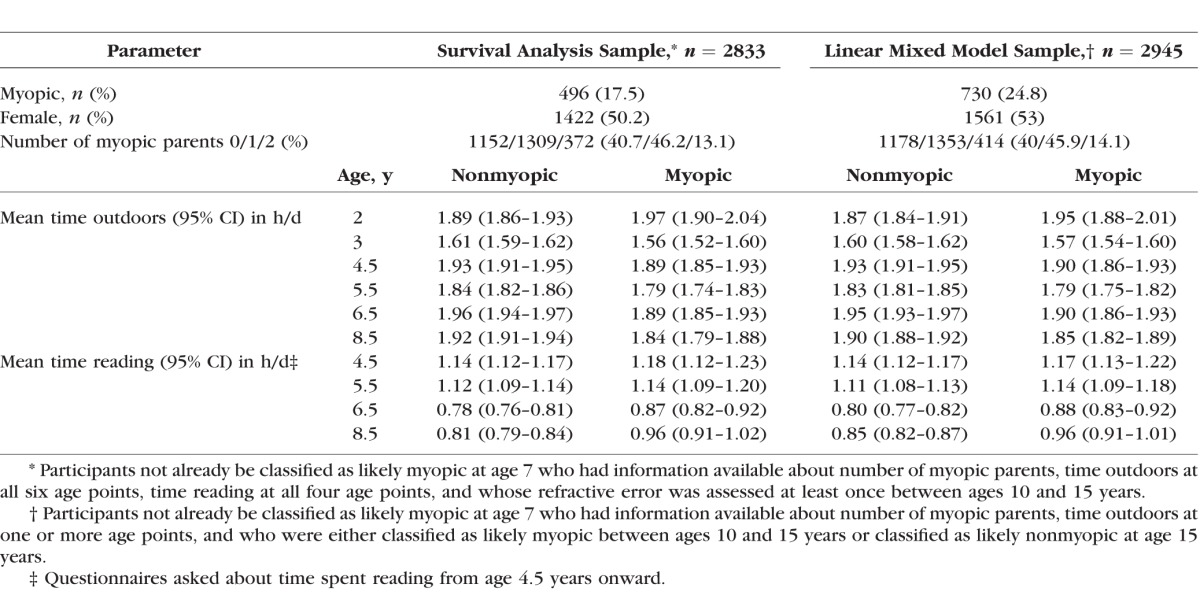
Time Spent Outdoors and Time Spent Reading in Groups of Participants Included in the Statistical Analyses

### Incident Myopia

**Figure 2:**
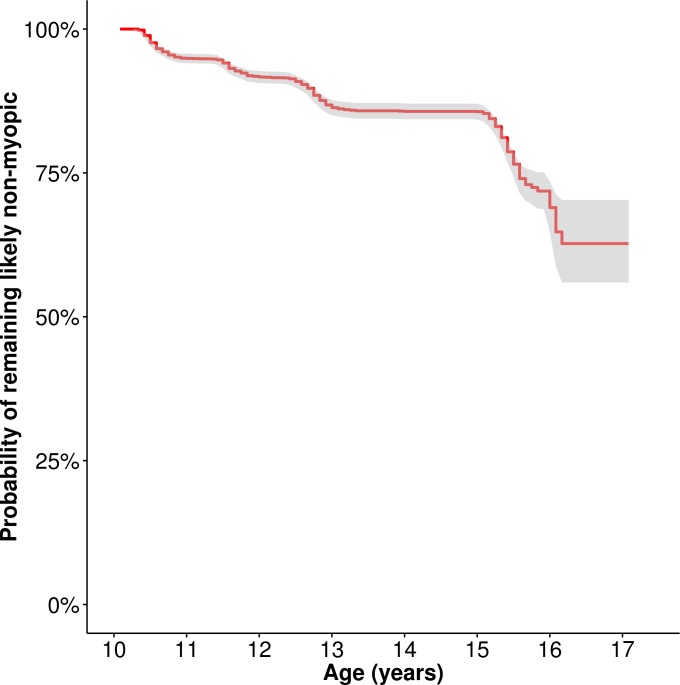
Kaplan-Meier survival curve (*n* = 2833). The line indicates the probability of remaining likely nonmyopic (i.e., surviving as a nonmyope). *Gray shading* denotes 95% CI.

Figure 2 shows a Kaplan-Meier survival curve for the 2833 children with complete covariate information who were not already classified as likely myopic before the age of 10 years. Between the ages of 10 and 15 years there was a steady increase in the number of children classified as likely myopic, resulting in a gradually reducing probability of a child remaining nonmyopic (Supplementary Table S3). The incidence of likely myopia in this sample over the 10- to 15-year observation period was 17.5%.

To address whether the amount of time spent outdoors in early childhood was associated with incident myopia during later childhood, HRs were calculated using Cox proportional hazards regression models (for the 2833 children with complete information for the covariates). Estimates of time spent outdoors were converted from units of hours per day to units of SDs (*z* scores) to scale the mean time outdoors at each age point to the same level and thereby emphasize child-to-child variation in behavior, and de-emphasize changes across childhood in the mean time spent outdoors by the whole cohort. At age 2 years, 1 SD unit corresponded very approximately to 50 minutes per day, whereas at older ages, 1 SD unit corresponded very approximately to 30 minutes per day. However, we stress that these estimates should be interpreted cautiously because they were inferred from categoric data.

**Figure 3:**
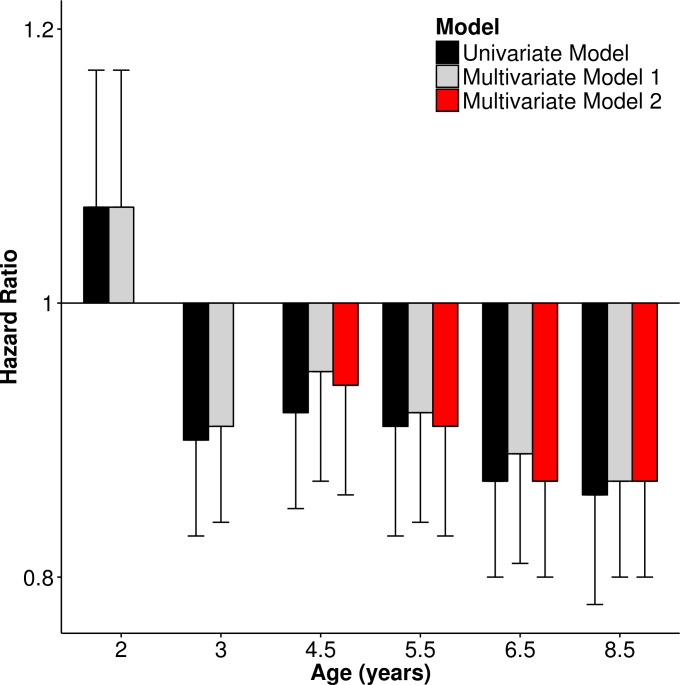
Hazard ratio for incident myopia between ages 10 and 15 years associated with a 1 SD change in time spent outdoors at specific earlier ages (*n* = 2833). Multivariate Model 1: adjusted for sex and number of myopic parents. Multivariate Model 2: adjusted for sex, number of myopic parents, and time spent reading. *Error bars* denote 95% CIs.

As shown in Figure 3, Table 2, and Supplementary Table S4, greater time outdoors at 2 years of age was associated with a subtle increase in the relative risk of incident myopia; however, this association had limited statistical support (*P* = 0.126). Beyond 2 years of age, greater time outdoors was associated with a reduction in the relative risk of incident myopia (the HR lay between 0.86 and 0.92 per 1 SD increase in time outdoors). Statistical support for these latter associations became increasingly strong with older age (*P* = 0.073 to *P* = 0.001). Across all questionnaire ages, the HRs for incident myopia were similar for the univariate and for two multivariate models that adjusted for sex and parental myopia, or for sex, parental myopia, and time spent reading. Analysis of Schoenfeld residuals under each model condition showed that the assumption of proportional hazards was met throughout. Thus, in summary, the amount of time children spent outdoors from ages 3 to 8 years was predictive of incident myopia in later childhood. Furthermore, these associations were entirely independent of the known risk factors for myopia: sex, parental myopia, and time spent reading.

**Table 2:**
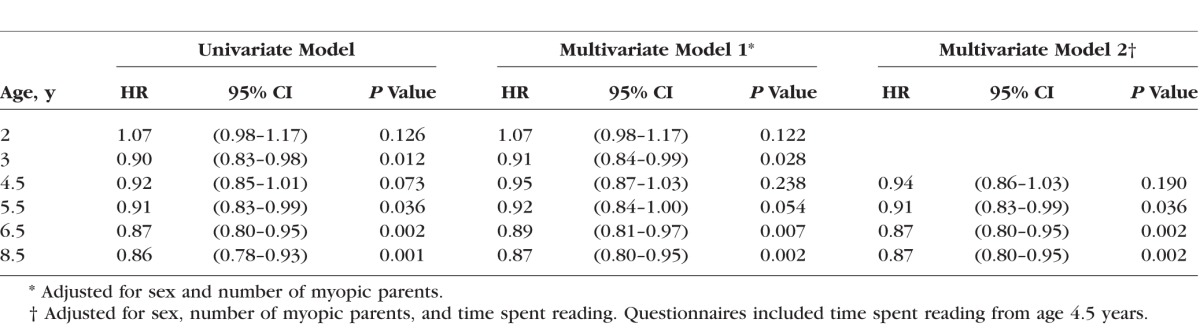
Hazard Ratio for Incident Myopia Between Ages 10 and 15 Years Associated With a 1 SD Per Day Increase in Time Spent Outdoors at Specific Earlier Ages (*n* = 2833)

### Correlations in Time Spent Outdoors at Different Ages

**Figure 4:**
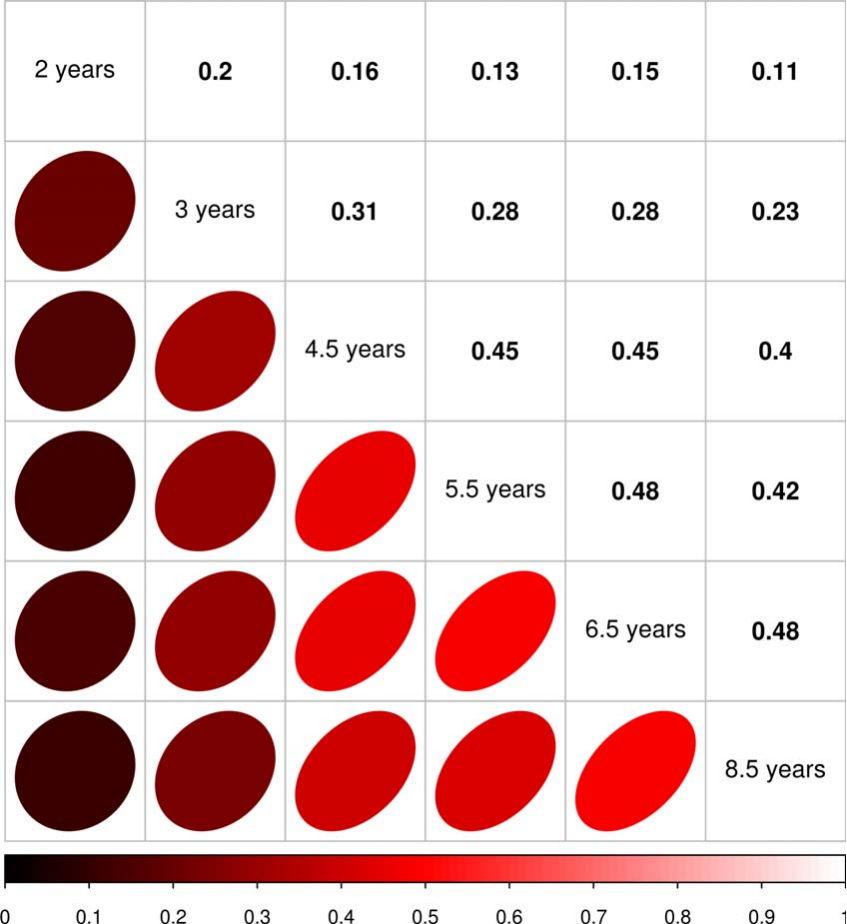
Pairwise Pearson correlation matrix of parentally reported time outdoors between ages 2.0 and 8.5 years (*n* = 2833).

The similar degree of association between incident myopia and time outdoors across the 3.0 to 8.5 years age range could potentially have arisen because one group of children were consistently spending more time outdoors than their peers throughout this period (i.e., those who experienced high outdoor exposure at earlier stages of childhood may have continued to have had high outdoor exposure as they got older). To explore this issue, a matrix of Pearson correlations between time outdoors at different ages was generated (Fig. 4). This suggested that children's behavior was moderately correlated between adjacent age points, but less closely correlated at more widely separated age points. For instance, the maximum correlation in time outdoors occurred between ages 6.5 and 8.5 years (*r* = 0.48), whereas for ages 3.0 and 8.5 years, the correlation was much lower (*r* = 0.11). Therefore, few individuals who spent a relatively high amount of time outdoors at younger ages continued to spend high amounts of time outdoors later in childhood. In other words, a single group of children was not responsible for the consistent association between time outdoors and incident myopia across childhood.

### Predictors of Time Outdoors Behavior

To take account of the correlated time outdoors behavior of individual children, linear mixed models were used to ascertain whether time spent outdoors at specific ages differed between those who did and did not become likely myopic in later childhood. For these models, all participants with time outdoors information available for at least one age point were eligible for inclusion. Additional inclusion criteria for the analysis were that the number of myopic parents was known, and that either the child was known to have developed likely myopia during the 10- to 15-year observation period, or else the child was seen at the last (15-year) clinic visit and found to be likely nonmyopic. Applying these criteria resulted in a sample size of 2945 participants (Table 1).

**Figure 5:**
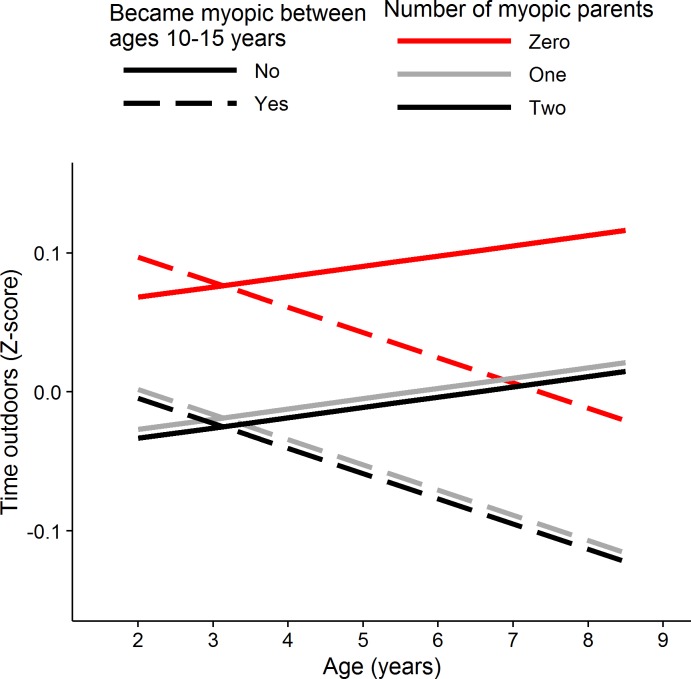
Best-fit model predictions of time spent outdoors in early childhood for children who did or did not become likely myopic during later childhood. Predictions from a linear mixed model designed to account for correlated time outdoors behavior across childhood for children who became likely myopic (*dashed lines*) or remained likely nonmyopic (*solid lines*), with colors denoting the number of myopic parents (0 = *red*, 1 = *gray*, 2 = *black*). By design, the average time outdoors per day at each age is zero. Positive *z* scores correspond to more time outdoors per day, negative *z* scores to less time outdoors.

Figure 5 shows the predicted time outdoors “trajectories” from the best-fitting linear mixed model for this sample. The parameter estimates of the best-fitting model are presented in Supplementary Table S5. The explanatory variables with the strongest association to time outdoors behavior were, first, the number of myopic parents and, second, being a child who would develop likely myopia in the future. Children with one or two myopic parents spent an average of approximately 0.1 SD units per day less time outdoors than children whose parents were both nonmyopic, with this effect being consistent in magnitude across the full 2.0- to 8.5-year age range. Future likely myopia had a more complex relationship with time spent outdoors, such that the predicted time outdoors trajectories between future likely myopes and nonmyopes gradually diverged across childhood (future myopia status × age interaction, *P* = 0.002). Thus, before age 4 years, there was little difference in time outdoors behavior between children who later became myopic compared with those who were to remain nonmyopic, whereas between the ages of 4.0 and 8.5 years, children who later became myopic spent progressively less time outdoors relative to their peers who remained nonmyopic, ending with an approximately 0.1 SD unit per day difference in time spent outdoors by age 8.5 years. There was suggestive evidence that girls spent slightly less time outdoors than boys (0.04 SD units per day, *P* = 0.14).

As a sensitivity analysis to examine the impact of the non-normal distribution of time outdoors *z* scores, an additional mixed model analysis was carried out in which time outdoors was categorized as an ordinal variable with four levels (Supplementary Fig. S2). Using a mixed model appropriate for the analysis of longitudinally collected ordinal data, similar results were obtained to those with the standard linear mixed model (Supplementary Table S6), implying that the results of the standard linear mixed model were robust to this source of potential bias.

## Discussion

A previous analysis^22^ of refractive development in ALSPAC participants had already reported that spending less time outdoors at age 8.5 years was predictive of an increased risk of developing myopia between ages 11.0 and 15.0 years. The results presented here build on this by showing that the association between time outdoors and incident myopia was not uniform across early childhood. Instead, greater time spent outdoors was associated with a trend toward a greater reduction in the risk of incident myopia for time spent outdoors at age 3.0 years through to age 8.5 years (Figs. 3, 5; future myopia status × age interaction, *P* = 0.002). At all ages, the relationship between time spent outdoors and incident myopia was independent of three other major risk factors for myopia: sex, parental myopia, and time spent reading. At its peak (age 8.5 years) the relative risk of incident myopia (HR) was 0.86 (95% confidence interval [CI] 0.78–0.93, *P* = 0.001) per 1 SD extra time outdoors per day. This figure differs from that reported previously^22^ in ALSPAC participants for time outdoors assessed at the same age point (HR = 0.66, 95% CI 0.56–0.78, *P* < 0.001), because in the latter study, time outdoors was modeled as a binary categoric variable (high versus low).

Our findings agree with previous work in suggesting that greater outdoor exposure is beneficial in protecting against myopia.^18,21,23–25,36,37^ We are not aware of any other studies that have comprehensively assessed the association between time outdoors and myopia over early childhood. However, in the CLEERE study, Zadnik et al.^38^ found that increased time outdoors was associated with a smaller reduction in the risk of incident myopia at age 6 years than at age 8 years. Specifically, the odds ratio for incident myopia was approximately 0.9 (95% CI 0.8–1.0) at age 6 and 0.8 (95% CI 0.7–0.9) at age 8, for each hour per day increase in time outdoors in the CLEERE study (note that the above figures were converted from units of hours per week^38^ to facilitate comparison with the ALSPAC findings).

Intervention trials have demonstrated a reduced incidence of myopia in children who were allocated to receive additional time outdoors in their daily school timetable relative to controls.^24,25,39^ However, for children who were already myopic, there was little if any effect of the intervention on reducing myopia progression.^24,25,39^ Thus, it remains an open question whether additional time outdoors should be recommended for slowing progression of existing myopia. Several biological mechanisms have been suggested to explain the association between increased outdoor exposure and reduced incident myopia (for reviews see Refs. 40–44). Of the two main theories, a role for vitamin D in modifying ciliary muscle tension and a role for enhanced secretion of dopamine in the retina in response to bright light, the evidence favors the theory involving dopamine.^45–48^ Light levels in the United Kingdom at noon vary, on average, from a low of approximately 15,000 lux in December to a high of approximately 60,000 lux in July.^49^

Independent of spending less time outdoors, ALSPAC participants who became likely myopic in later childhood spent more time reading at specific ages in early childhood (Table 1). This association between time spent reading at age 8.5 years and incident myopia was reported in the earlier ALSPAC study mentioned above.^22^ Here, we found that the association was also present at age 6.5 years, with those who later became likely myopic spending 0.11 (0.03–0.19) SD units *more* time reading than average per day, and those who did not become likely myopic spending 0.04 (−0.01 to 0.08) SD units *less* time reading per day (HR for each 1 SD unit of additional time reading per day = 1.12, 95% CI 1.03–1.22, *P* = 0.005; *n* = 2833). Time reading when the participants were aged 4.5 and 5.5 years was not associated with future myopia (*P* = 0.45 and *P* = 0.49, respectively). Interestingly, in the CLEERE longitudinal study of refractive development, time reading was not predictive of incident myopia and, instead, reading behavior diverged between myopes and nonmyopes only after myopia onset.^21^ One difference that may have contributed to the contrasting conclusions regarding whether time reading was (ALSPAC) or was not (CLEERE) predictive of incident myopia was that in the CLEERE study, the analysis model considered the number of years preceding myopia onset rather than the age of participants per se. If differences in reading behavior are highly age-dependent, as our results suggested, then analyzing groups of children of varying age may have masked any small difference in reading behavior. The Singapore Cohort of the Risk factors for Myopia (SCORM) study, which examined refractive development of children living in Singapore, also found no association between time reading and incident myopia. Key differences between SCORM and the current study that may have contributed to the different findings were that approximately 30% of SCORM participants were already myopic at the baseline age of 7 years (compared with ∼2% in ALSPAC) and the shorter follow-up period (3 vs. 8 years).

We speculate that parental influence contributed to the different ages at which time outdoors and time reading became predictive of future myopia in ALSPAC participants. At age 4 to 6 years, children may not do much reading on their own, but with their parents, and therefore parental behavior would be very important at these early ages. A child's own reading behavior is likely to emerge at later ages, and this might differ from that of their parents. An analogous pattern of gradually reducing parental influence is likely to exist regarding time spent outdoors; however, the child's own behavior may become established earlier for time outdoors than for time reading.

Our study had several limitations. First, the lack of cycloplegia during autorefraction in children is known to reduce the precision of refractive error measurement, and to introduce a bias toward more negative values.^28^ Both the degree of measurement error and the level of “over-minus” bias is worse the younger the child.^50^ For participants in the ALSPAC study, the noncycloplegic autorefraction threshold of −1.00 D used here to classify individuals as likely myopic was reported previously^22,51^ to have sensitivity of 0.91 and specificity of 0.92 when carried out at age 15 years, and sensitivity of 0.67 and specificity of 0.95 when carried out at age 7 years, in correctly detecting myopes (true refractive error ≤ −0.75 D). This bias and measurement error resulting from the lack of cycloplegia will have resulted in the misclassification of some children as myopic/nonmyopic and vice versa, especially at younger ages. The misclassification, in turn, will have reduced the statistical power of our analyses to detect associations between time outdoors exposure and incident myopia, and could have caused the magnitude of any associations to be either under- or overestimated. However, the use of survival analysis (rather than a method focussing on a single time point) will have mitigated measurement error effects to some extent, because those children wrongly classified as nonmyopic at an early age are likely to have been correctly classified as myopic when tested at an older age (both because myopia tends to progress with age, and because noncycloplegic autorefraction becomes more precise with age). As fewer than 20% of children were categorized as developing likely myopia during the observation period, the chosen threshold of −1.00 D appeared relatively stringent; that is, estimates of likely myopia may have been inflated to a greater extent if a threshold of −0.50 or −0.75 D had been adopted.

Second, information about time spent outdoors and time reading was obtained from responses to questionnaires completed by the mothers/primary carers. Although these responses would not have been subject to major recall bias, questions about summer and winter activities were posed in the same questionnaire, meaning that recall bias may not have been excluded entirely. Third, the questionnaires lacked fine-grain detail in the response options available, and had an upper limit for time spent outdoors and time reading of “3 or more hours.” Therefore, it was not possible to ascertain the precise amount of time children spent outdoors, especially when this was beyond 3 hours. Recent studies have tried to overcome this type of limitation by using miniaturized sensors to accurately and quantitatively record light intensity exposures during children's day-to-day activities.^36,52,53^ Fourth, questions about the time children spent outdoors were not included in ALSPAC questionnaires beyond the age of 8.5 years. Therefore, we could not determine whether time spent outdoors at older ages continued to have an increasingly strong association with incident myopia. Finally, not all children attended every clinical visit and information about the number of myopic parents was missing for a high proportion of the cohort (Supplementary Table S7).

Strengths of this study were that information about time spent outdoors was ascertained during early childhood (age 2.0–8.5 years), whereas incident myopia was monitored during later childhood (age 10–15 years). Because these two periods did not overlap, reverse causality is very unlikely to have driven the observed association (i.e., it is unlikely that children spent less time outdoors because they wore glasses to correct myopia). Also, as the ALSPAC cohort was initially a population-based sample, and was designed to investigate a wide range of health and well-being measures, selection bias should have been lower for this study than for studies investigating ocular traits specifically,^38,54,55^ in which participants with refractive errors may have been more likely to attend follow-up visits than nonmyopes.

In conclusion, in this large, population-based birth cohort, time spent outdoors between ages 3.0 and 8.5 years was inversely associated with incident myopia in later childhood. There was no evidence to suggest that the association between time outdoors and incident myopia was mediated by children spending less time reading, or by virtue of having myopic parents (note that although children with one or two myopic parents did tend to spend less time outdoors, the magnitude and statistical support for the time outdoors versus myopia association was unaffected by adjusting for parental myopia). There was evidence that the reduced risk of incident myopia associated with greater time outdoors increased progressively through to age 8.5 years (*P* = 0.002; Supplementary Table S5). However, the magnitude of this increase was modest (less than 2-fold) and, in general, across the full 3.0- to 8.5-year age range, each additional 1 SD unit of time spent outdoors per day was associated with an approximately 10% reduced risk of incident myopia. Coupled with the results of RCTs for time spent outdoors,^24,25^ our findings provide an evidence base for recommending children to spend more time outdoors across the full 3.0- to 8.5-year age range so as to reduce the risk of incident myopia (note that additional time outdoors at older ages also may be beneficial; however, our study did not investigate ages beyond 8.5 years). Interventions aiming to reduce the incidence of myopia by providing a specific duration of extra time outdoors per day (e.g., 1 extra hour) may have greater impact when targeting children aged 5.5 to 8.5 years than when targeting younger ages.

## Supplementary Material

Supplement 1
