# Supplementary material for: Time Outdoors at Specific Ages During Early Childhood and the Risk of Incident Myopia
Source: Invest Ophthalmol Vis Sci. 2017 Feb;58(2):1158–66. doi: 10.1167/iovs.16-20894 (PMC5341622; doi:10.1167/iovs.16-20894)
Supplement: Supplement 1 [file iovs-58-02-26_s01.pdf]

SUPPLEMENTARY MATERIAL

Time outdoors at specific ages during early childhood and the risk of incident myopia

Rupal L. Shah[1], Yu Huang[1,2], Jeremy A. Guggenheim[1] and Cathy Williams[3]

[1] School of Optometry & Vision Sciences, Cardiff University, Cardiff, UK.

[2] School of Optometry, Hong Kong Polytechnic University, Kowloon, Hong Kong.

[3] School of Social and Community Medicine, University of Bristol, Bristol, UK.

Index	Page
Supplementary Methods	
Inference of parental refractive error from questionnaire responses	2
Linear mixed model analyses	3
Supplementary Tables	
Table S1. Time spent outdoors and time spent reading questions asked in questionnaires	4
Table S2. Time spent outdoors and time spent reading in groups of participants included in the statistical analyses (coded as Z-scores)	6
Table S3. Life table for incident myopia in participants with full information on covariates	7
Table S4. Hazard ratio (HR) for incident myopia associated with a 1 hour/day increase in time spent outdoors at specific ages	7
Table S5. Best fit linear mixed model for predicting time outdoors behaviour across the age range 2 to 8.5 years (outcome variable), in children who later in childhood either did or did not become likely myopic	8
Table S6. Best fit "mixor" mixed model for predicting time outdoors behaviour across the age range 2 to 8.5 years (outcome variable), in children who later in childhood either did or did not become likely myopic	8
Table S7. The number of participants with missing covariate information	9
Supplementary Figures	
Figure S1. Inferring maternal refractive error from questionnaire responses	10
Figure S2. Frequency distribution of the time outdoors variable for the children included in the mixed model analyses	11
Supplementary references	12

Supplementary Methods

Inference of parental refractive error from questionnaire responses.

When time permitted after the assessment of ALSPAC children's refractive error and visual function at the 15-year research clinic, the accompanying parent was invited to undergo non-cycloplegic autorefraction. The child's mother accompanied the child more often than the father. During their pregnancy, mothers were asked the following question (for each eye separately): "How would you rate your sight without glasses?" Response options were: "always very good", "I can't see clearly at a distance", "I can't see clearly close up" and "I can't see much at all". There were 1609 mothers with information available for both autorefraction and questionnaire responses. Plots for these data (Supplementary Figure S1) indicated that the responses, "always very good" and "I can't see clearly close up" were associated with non-myopic refractive errors, while the responses, "I can't see clearly at a distance" and "I can't see much at all" were associated with myopic refractive errors.

Fathers were asked the same set of questions about their eyesight. Hence, all parents who provided questionnaire responses about their eyesight were classified as follows. If the response for both eyes was, "I can't see clearly at a distance" or "I can't see much at all", or a combination of these two responses, the parent was classed as being myopic. Parents with responses for both eyes of "always very good" or "I can't see clearly close up" or a combination of these two responses were classed as being non-myopic. Any other combination of responses resulted in the parent's inferred refractive error being set as "missing". This classification scheme differed from that used in previous studies[1,2] of refractive development in ALSPAC participants (formerly, parents were classified as myopic if they answered ''I can't see clearly at a distance'' for both eyes, and as non-myopic otherwise, which led to greater numbers of missing data).

Linear mixed model analyses.

Linear mixed models with an autoregressive correlation structure were fitted using the equation:

$$\text{Time Outdoors}_{ij} \sim \text{N}(XB,\ \Omega)$$

$$\text{Time Outdoors}_{ij} = \beta_{0ij}\text{Constant} + \beta_{1j}\text{Age}_{ij} + \beta_{2}\text{Myopic}_{j} + \beta_{3}\text{Sex}_{j} + \beta_{4}\text{NMP1}_{j} + \beta_{5}\text{NMP2}_{j} + \beta_{6}\text{Age x Myopic}_{ij} + \beta_{7}\text{Age x Sex}_{ij}$$

$$\beta_{0ij} = \beta_0 + u_{0j} + e_{0ij}$$

$$\beta_{1j} = \beta_1 + u_{1j}$$

$$\begin{bmatrix} u_{0j} \\ u_{1j} \end{bmatrix} \sim \text{N}(0,\ \Omega_u) \ : \ \Omega_u = \begin{bmatrix} \sigma^2_{u0} & \\ \sigma_{u01} & \sigma^2_{u1} \end{bmatrix}$$

$$\begin{bmatrix} e_{0ij} \end{bmatrix} \sim \text{N}(0,\ \Omega_e) \ : \ \Omega_e = \begin{bmatrix} \sigma^2_{e0} \end{bmatrix}$$

where,

Time Outdoors$_{ij}$ is the time outdoors Z-score of participant *j* at age *i*,

β_0 is the mean time outdoors Z-score for the population from which the participants are drawn,

Age$_{ij}$ is the age from baseline (2 years-old), in years, of participant *j*,

Sex$_j$ is a binary variable indicating the gender of participant *j (0=male, 1=female),*

Myopic$_j$ is a binary variable indicating the likely myopia status of participant *j (0=likely non-myopic, 1=likely myopic),*

NMP1$_j$ is a binary variable indicating if participant *j* has 1 myopic parent *(No=0, Yes=1),*

NMP2$_j$ is a binary variable indicating if participant *j* has 2 myopic parents *(No=0, Yes=1),*

e_{0ij} is the residual for participant *j* at visit *i*,

Ω represents the variances and covariances of the random terms in the model, which are assumed to be drawn from normal distributions with means of zero.

σ_{u0} equals var(u_0), the variance in the random intercepts,

σ_{u1} equals var(u_1), the variance in the random slopes,

σ_{u01} equals covar(u_{01}), the covariance between the random intercepts and slopes,

σ^2_{e0} equals var(e_0), the variance of the residuals.

Supplementary Table S3. Time spent outdoors and time spent reading questions asked in questionnaires. The last column shows how categorical response options were converted to quantitative units (hours per day).

Age of child	Questions asked of mother/primary carer	Response options for each question	Time (in hours/day) assigned to response option
**2 years**	Hours per Week child spends outside	None at all<br>One to two hours<br>Three to six hours<br>Seven to thirteen hours<br>Fourteen to twenty hours<br>Twenty-one or more hours	0<br>1.5/7<br>4.5/7<br>10.5/7<br>17.5/7<br>21/7
**3 years**	Hours per Day Outside on a Weekday<br>Hours per Day Outside on a Weekend day	None at all<br>Less than one hour<br>One to two hours<br>Two or more hours	0<br>0.5<br>1.5<br>2
**4.5 years**	Hours per Day Outside in Summer on Weekday<br>Hours per Day Outside in Summer on Weekend Day<br>Hours per Day Outside in Winter on Weekday<br>Hours per Day Outside in Winter on Weekend Day<br>Hours per Day Looking at Books on Weekday<br>Hours per Day Looking at Books on Weekend Day	None at all<br>Less than one hour<br>One to two hours<br>Three or more hours	0<br>0.5<br>1.5<br>3
**5.5 years**	Hours per Day Outside in Summer on Weekday<br>Hours per Day Outside in Winter on Weekday<br>Hours per Day Looking at Books on Weekday<br>Hours per Day Outside in Summer on Weekend day<br>Hours per Day Outside in Winter on Weekend day<br>Hours per Day Looking at Books on Weekend day	None at all<br>Less than one hour<br>One to two hours<br>Three or more hours	0<br>0.5<br>1.5<br>3

Age of child	Questions asked of mother/primary carer	Response options for each question	Time (in hours/day) assigned to response option
**6.5 years**	Time per Day Outside in Summer on Weekday<br>Time per Day Outside in Winter on Weekday<br>Hours per Day Reading Books on Weekday<br>Hours per Day Outside in Summer on Weekend day<br>Time per Day Outside in Winter on Weekend day<br>Hours per Day Reading Books on Weekend day<br>Hours per Day Outside in Summer during Holidays<br>Hours per Day Outside in Winter during Holidays<br>Hours per Day Reading Books during Holidays	None at all<br>Less than one hour<br>One to two hours<br>Three or more hours	0<br>0.5<br>1.5<br>3
**8.5 years**	Time per Day Outside in Summer on Weekday<br>Time per Day Outside in Winter on Weekday<br>Hours per Day Reading Books on Weekday<br>Time per Day Outside in Summer on Weekend day<br>Time per Day Outside in Winter on Weekend day<br>Hours per Day Reading Books on Weekend day<br>Hours per Day Outside in Summer during Holidays<br>Hours per Day Outside in Winter during Holidays<br>Hours per Day Reading Books during Holidays	None at all<br>Less than one hour<br>One to two hours<br>Three or more hours	0<br>0.5<br>1.5<br>3

Supplementary Table S4. Time spent outdoors and time spent reading in groups of participants included in the statistical analyses (as in Table 1 but with time outdoors and time reading coded as Z-scores).

		Survival analysis sample[a] (N = 2833)		Linear mixed model sample[b] (N = 2945)	
	Age (years)	Non-myopic	Myopic	Non-myopic	Myopic
Mean time outdoors (95% CI) in units of standard deviations per day	2	-0.016 (-0.056 to 0.025)	0.073 (-0.012 to 0.158)	-0.021 (-0.064 to 0.022)	0.065 (-0.009 to 0.138)
	3	0.022 (-0.017 to 0.062)	-0.106 (-0.202 to -0.009)	0.017 (-0.025 to 0.059)	-0.052 (-0.132 to 0.027)
	4.5	0.016 (-0.024 to 0.057)	-0.077 (-0.169 to 0.014)	0.018 (-0.026 to 0.061)	-0.054 (-0.131 to 0.024)
	5.5	0.019 (-0.021 to 0.059)	-0.089 (-0.179 to 0.000)	0.022 (-0.021 to 0.066)	-0.067 (-0.146 to 0.012)
	6.5	0.030 (-0.010 to 0.070)	-0.140 (-0.232 to -0.048)	0.031 (-0.013 to 0.075)	-0.094 (-0.175 to -0.012)
	8.5	0.035 (-0.005 to 0.075)	-0.163 (-0.255 to -0.072)	0.025 (-0.019 to 0.069)	-0.077 (-0.157 to 0.003)
Mean time reading (95% CI) in units of standard deviations per day	4.5	-0.010 (-0.050 to 0.031)	0.045 (-0.043 to 0.133)	-0.012 (-0.056 to 0.031)	0.038 (-0.040 to 0.115)
	5.5	-0.008 (-0.048 to 0.033)	0.037 (-0.052 to 0.125)	-0.012 (-0.055 to 0.032)	0.035 (-0.042 to 0.112)
	6.5	-0.028 (-0.068 to 0.012)	0.132 (0.038 to 0.226)	-0.036 (-0.079 to 0.007)	0.110 (0.027 to 0.194)
	8.5	-0.045 (-0.085 to -0.006)	0.212 (0.116 to 0.309)	-0.047 (-0.090 to -0.004)	0.145 (0.062 to 0.227)

[a] Participants not already be classified as likely myopic at age 7 who had information available about number of myopic parents, time outdoors at all 6 age-points, time reading at all 4 age-points, and whose refractive error was assessed at least once between age 10-15 years.

[b] Participants not already be classified as likely myopic at age 7 who had information available about number of myopic parents, time outdoors at 1 or more age-points, and who were either (a) classified as likely myopic between age 10-15 years or (b) classified as likely non-myopic at age 15 years.

[c] Questionnaires asked about time spent reading from age 4.5 years onwards.

Supplementary Table S3. Life table for incident myopia in participants with full information on covariates (N = 2833).

Age (years)	Number entering observation period	Number becoming myopic during observation period	Percentage remaining non-myopic	95% CI for Percentage remaining non-myopic
10-11	2833	142	0.950	(0.941 to 0.957)
11-12	2571	86	0.918	(0.907 to 0.928)
12-13	2352	125	0.869	(0.856 to 0.881)
13-14	1763	13	0.863	(0.849 to 0.875)
14-15	1680	1	0.862	(0.849 to 0.875)
15-16	1661	125	0.797	(0.781 to 0.813)
16-17	49	4	0.732	(0.663 to 0.790)

Supplementary Table S4. Hazard ratio (HR) for incident myopia associated with a 1 hour/day increase in time spent outdoors at specific ages (N = 2833). These results are the same as those presented in Table 1 but with time outdoors and time reading re-coded.

Age (years)	Univariate Model			Multivariate Model 1 [a]			Multivariate Model 2 [b]		
	Hazard Ratio	95% CI	P	Hazard Ratio	95% CI	P	Hazard Ratio	95% CI	P
**2**	1.08	(0.98 to 1.20)	0.126	1.09	(0.98 to 1.21)	0.122			
**3**	0.77	(0.63 to 0.94)	0.012	0.79	(0.65 to 0.98)	0.028			
**4.5**	0.84	(0.70 to 1.02)	0.073	0.89	(0.74 to 1.08)	0.238	0.88	(0.73 to 1.06)	0.190
**5.5**	0.82	(0.68 to 0.99)	0.036	0.83	(0.69 to 1.00)	0.054	0.81	(0.67 to 0.99)	0.036
**6.5**	0.72	(0.58 to 0.89)	0.002	0.75	(0.60 to 0.92)	0.007	0.72	(0.58 to 0.89)	0.002
**8.5**	0.71	(0.58 to 0.86)	0.001	0.74	(0.61 to 0.90)	0.002	0.73	(0.60 to 0.89)	0.002

[a] Adjusted for sex and number of myopic parents.

[b] Adjusted for gender, number of myopic parents, and time spent reading. Questionnaires included time spent reading from age 4.5 years.

Supplementary Table S5. Best fit linear mixed model for predicting time outdoors behaviour across the age range 2 to 8.5 years (outcome variable), in children who later in childhood either did or did not become likely myopic (N = 2945).

Model explanatory variables	Beta [a]	95% CI	P
Intercept	0.068	(0.018 to 0.118)	0.007
Age (years)	0.007	(-0.001 to 0.015)	0.073
Myopic (referent non-myopic)	0.029	(-0.039 to 0.096)	0.401
Gender (referent male)	-0.036	(-0.082 to 0.011)	0.135
1 Myopic parent (referent 0 myopic parents)	-0.095	(-0.146 to -0.045)	<0.001
2 Myopic parents (referent 0 myopic parents)	-0.102	(-0.175 to -0.028)	0.007
Myopic x Age interaction	-0.025	(-0.042 to -0.009)	0.002

[a] Change in time outdoors (in units of standard deviation) per unit change of predictor variable, e.g. 1 year.

Supplementary Table S6. Best fit "mixor" mixed model for predicting time outdoors behaviour across the age range 2 to 8.5 years (outcome variable), in children who later in childhood either did or did not become likely myopic (N = 2945). Parameters obtained from a mixed model analysis with time outdoors categorised as an ordinal variable with 4 levels, analysed using "mixor". Note the similar findings to those above for the standard linear mixed model (Table S5).

Model explanatory variables	Beta [a]	95% CI	P
Intercept	1.893	(1.774 to 2.012)	<0.001
Age (years)	-0.091	(-0.105 to -0.076)	<0.001
Myopic (referent non-myopic)	0.056	(-0.103 to 0.215)	0.492
Gender (referent male)	-0.091	(-0.194 to 0.012)	0.083
1 Myopic parent (referent 0 myopic parents)	-0.236	(-0.348 to -0.124)	<0.001
2 Myopic parents (referent 0 myopic parents)	-0.239	(-0.397 to -0.082)	0.003
Myopic x Age interaction	-0.045	(-0.074 to -0.016)	0.002

Change in time outdoors (in units of standard deviation) per unit change of predictor variable, e.g. 1 year.

Supplementary Table S7. The number of participants with missing covariate information

Covariate	Number of participants with refractive error information at 1 or more age-points	Number of participants with missing information for covariate (%)
Number of myopic parents	9095	4034 (44.4%)
Time outdoors (age 2 years)	9095	1592 (17.5%)
Time outdoors (age 3 years)	9095	1751 (19.3%)
Time outdoors (age 4.5 years)	9095	2021 (22.2%)
Time outdoors (age 5.5 years)	9095	2156 (23.7%)
Time outdoors (age 6.5 years)	9095	2470 (27.2%)
Time outdoors (age 8.5 years)	9095	2281 (25.1%)
Time reading (age 4.5 years)	9095	1982 (21.8%)
Time reading (age 5.5 years)	9095	2121 (23.3%)
Time reading (age 6.5 years)	9095	2445 (26.9%)
Time reading (age 8.5 years)	9095	2236 (24.6%)

Supplementary Figure S1. Inferring maternal refractive error from questionnaire responses. Maternal questionnaire response options were: (A) Always very good, (B) I can't see clearly at a distance, (C) I can't see clearly close up, and (D) I can't see much at all. Refractive error was assessed by non-cycloplegic autorefraction. Boxes show the position of the 25th and 75th percentiles; whiskers show 1.5 times the inter-quartile range (IQR).

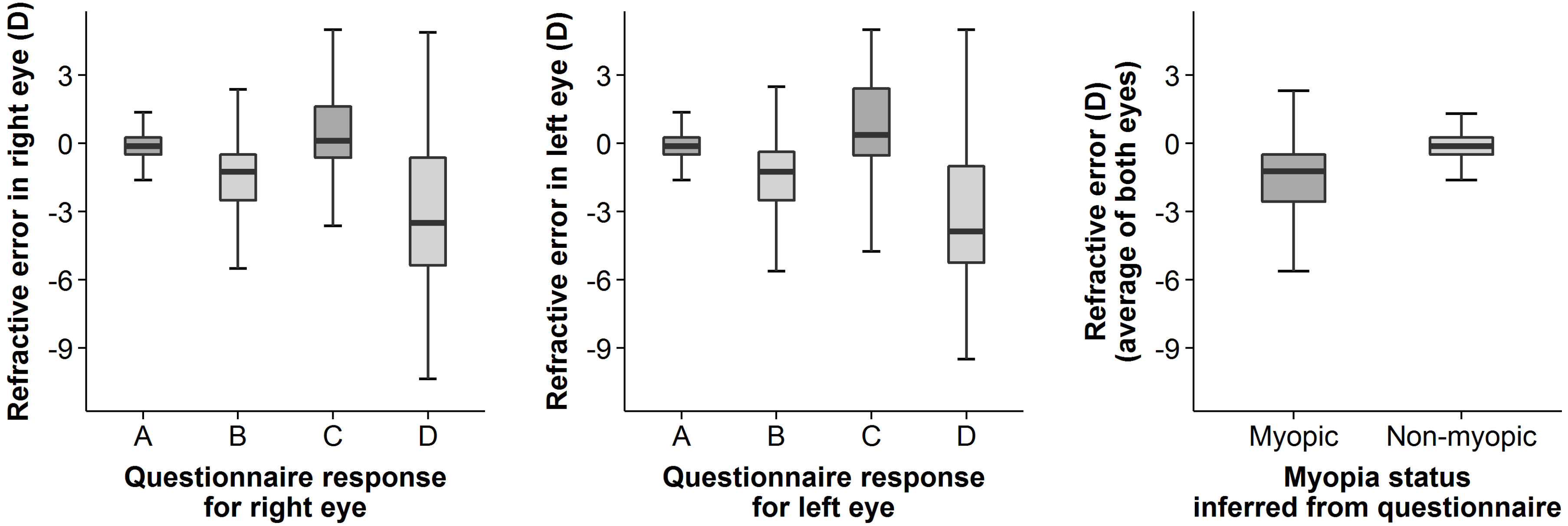

Supplementary Figure S2. Frequency distribution of the time outdoors variable for the children included in the mixed model analyses (N = 2945). Panels **a-f**, distribution of time outdoors Z-scores (x-axis) at each age point, as used in the linear mixed model analysis. Panels **g-l**, distribution of time outdoors ordinal category (x-axis) at each age point used in the “mixor” analysis. Note that the Z-score distributions are non-normal, and that the variable takes one of only a few values at early ages.

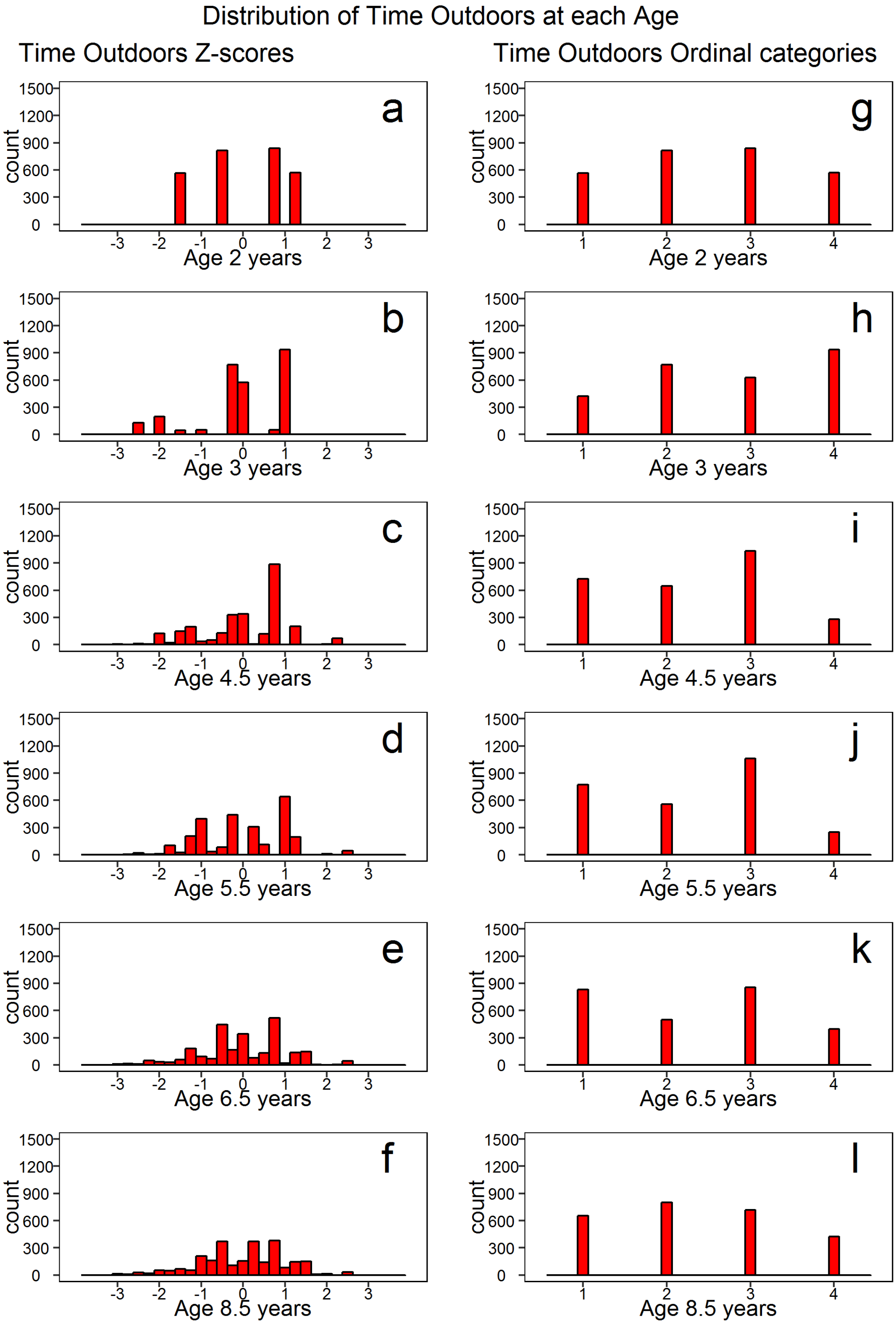

Supplementary References

1 Guggenheim, J. A. *et al.* Time outdoors and physical activity as predictors of incident myopia in childhood: a prospective cohort study. *Invest Ophthalmol Vis Sci* **53**, 2856-2865 (2012).

2 Guggenheim, J. A. *et al.* Does vitamin D mediate the protective effects of time outdoors on myopia? Findings from a prospective birth cohort. *Invest Ophthalmol Vis Sci* **55**, 8550-8558 (2014).
